# Posterior pole asymmetry analysis and retinal nerve fibre layer thickness measurements in primary angle-closure suspect patients

**DOI:** 10.1186/s12886-019-1034-0

**Published:** 2019-01-28

**Authors:** Yi Zha, Wei Huang, Jinfei Zhuang, Jianqiu Cai

**Affiliations:** 1 Department of Ophthalmology, The Second Affiliated Hospital and Yuying Children’s Hospital of Wenzhou Medical University, Wenzhou, 325027 Zhejiang China; 2 Department of Ophthalmology, The first People’s Hospital of Shaoyang, Shaoyang, 422000 Hunan China

**Keywords:** Primary angle-closure suspects, Optical coherence tomography, Retinal nerve fiber layer, Posterior pole asymmetry analysis

## Abstract

**Purpose:**

To measure peripapillary retinal nerve fiber layer (RNFL) thickness and posterior pole retinal thickness in primary angle-closure suspects (PACS) by Spectral domain optical coherence tomography (SD-OCT) and to be compared with normal subjects.

**Methods:**

Thirty five primary angle-closure suspect patients and thirty normal subjects were enrolled in this study. Peripapillary RNFL and posterior pole retinal thickness by posterior pole asymmetry analysis (PPAA) in SD-OCT were measured.

**Results:**

No significant difference was found in both groups on age, sex distribution, refractive error, intraocular pressure (IOP) and axial length. The PACS group exhibited significantly thinner macular retinal thickness and larger asymmetry on posterior pole region compared with the control group. Yet no significant difference of peripapillary RNFL parameters was found between PACS group and normal control group. A negative correlation was observed between the total retinal thickness on posterior pole region and age when all the PACS participants were analyzed.

**Conclusions:**

Posterior pole retinal thickness measurements obtained by Heidelberg Spectralis SD-OCT using PPAA showed significant thinner change in PACS group than healthy controls. Only age seemed to be an indicator in the occurrence of glaucomatous damage in PACS patients.

## Background

Glaucoma is one of the main causes of eye blindness in the world [1]. In Asia, the most common type of glaucoma is primary angle-closure glaucoma (PACG) [2, 3]. Primary angle-closure disease (PACD) causes half of glaucoma-induced blindness [4]. PACD is made up three types: primary angle-closure suspect (PACS), PACG and primary angle closure (PAC) as well [5, 6]. Study has showed that about 22% of PACS could be developed to PAC and 28% of the latter advanced to PACG [6, 7]. Since visual field is found defected only after 40–50% of RGCs has lost, it is hard to diagnose the glaucoma early by visual field.

The diagnosis of PACS is quite difficult because patients with PACS always do not exhibit apparent sign such as elevated intraocular pressure (IOP), visual field loss or any degree of peripheral anterior synechia. Posterior pole asymmetry analysis (PPAA), a new OCT software developed for glaucoma, was found to increase diagnostic accuracy of glaucoma [8]. The purpose of our study was to detect PACS patients’ posterior pole retinal thickness and peripapillary retinal nerve fiber layer (RNFL). We hypothesize that PACS patients have fewer RGCs in posterior pole region and might therefore show a thinner retinal thickness in PPAA. We hope it will be a new diagnostic technique showing some early defects in PACS patients.

## Methods

This study was conducted by the Declaration of Helsinki and approved by the ethics committee of the 2nd affiliated hospital of Wenzhou Medical University which belonged to Wenzhou Medical University. All participates involved in the study were required to sign written informed consent.

### Patients

All PACS subjects were enrolled from hospital’s outpatient clinic. The healthy subjects were collected from our Examination Center. All subjects were included in this study only when they met the following requirement: corrected distance vision acuity(subjective refraction) of ≧ 0.5 Snellen, refractive error of ≤ ± 5.00 diopters (D) spherical, and ≤ ± 3.00 D cylindrical, and age between 40 years and 70 years. PACS has no peripheral anterior synechiae but IOP of lower than 21 mmHg, normal visual field and regular disc morphology, with the non-visibility of the filtering portion of trabecular meshwork for > 180° on gonioscopy [9]. The subjects were excluded in the study if they had any history of ocular disease or intraocular surgery, fixation losses and signal-to-noise ratio of lower than 16. Only one eye were included randomly in the research if both eyes of the subjects met the inclusion criteria.

All subjects had to complete a set of examinations including best corrected visual acuity (BCVA), auto-refraction, slit lamp, gonioscope, IOP(Goldmann applanation tonometry), visual field(Haag Streit AG, Könitz, Switzerland), and OCT measurement.

### OCT imaging acquisition

**Fig. 1:**
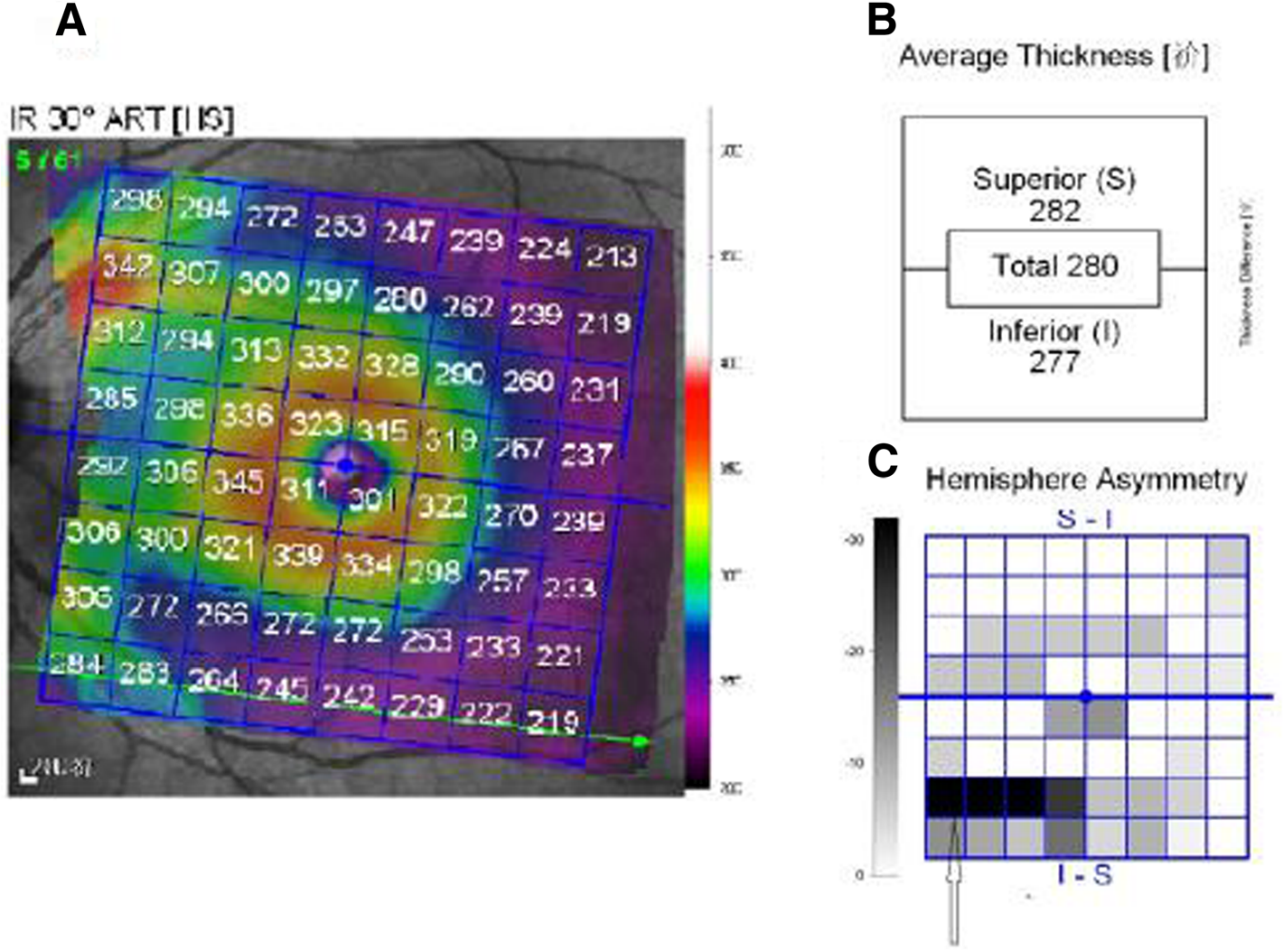
Posterior pole thickness asymmetry analysis of Spectralis OCT. **a** Color-coded thickness map for each eye that represent retinal thickness patterns. **b** the mean macular thickness of total, superior and inferior regions; **c** A gray-scale grid (labeled OS-OD asymmetry) represents inter eye thickness asymmetry

The same experienced examiner finished all OCT measurement (Heidelberg Engineering, Heidelberg, Germany). Non-mydriatic eyes were used for OCT examination. Only images of good quality of OCT were used for further analysis. The PPAA screen showed the mean superior, inferior and total retinal thickness on posterior pole region (Fig. 1). The mean retinal thickness asymmetry(RTA) was calculated for all cells of the posterior pole grid between superior and inferior hemispheres retinal thickness of the same eye. In this study, the central 4 cells of the whole 64 squares positioned around the fovea was named as the central macular area (called “region 1”), whereas the surrounding 16 square around the region 1 was named as the peri-central area (called “region 2”), the surrounding 20 square areas around region 2 as the peri-macular area (called “region 3”), and the outer 28 square areas as the peripheral area (called “region 4”). The average thickness of each region was calculated as well.

**Fig. 2:**
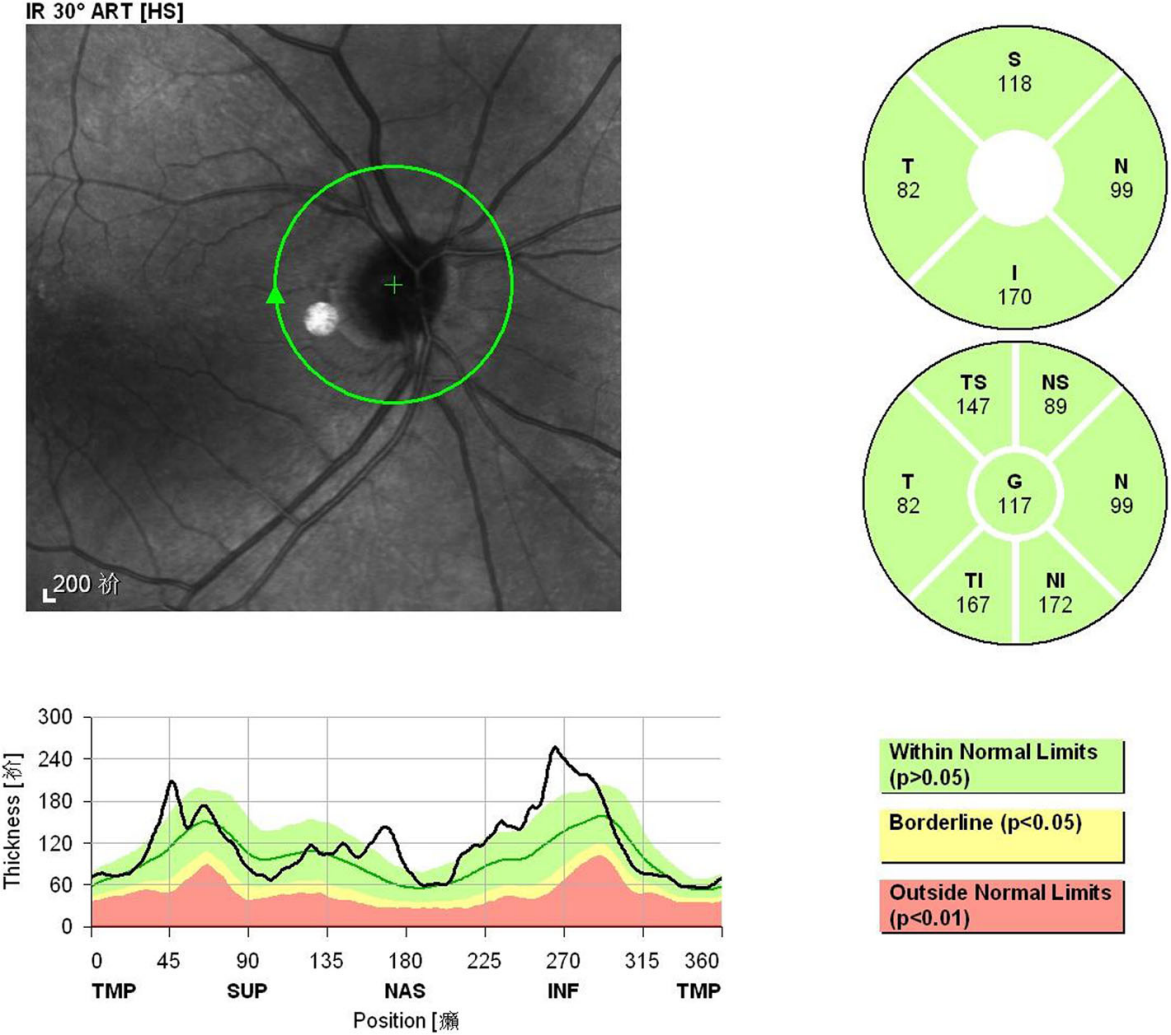
Quantitative portion of peripapillary RNFL thickness report for participates. The report provides quantitative data for each eye, dividing peripapillary RNFL thickness data into 4-sector and 7-sector circular graphs. For this study, we used the global thickness (G) value from the 7-sector output graph; the superior (S), inferior (I),nasal(N) and temporal(T) thickness values from the 4-sector output graph; and the other supero-nasal(NS), supero-tempora(TS), infero-temporal(TI) and infero-nasal(NI) from the 7-sector output graph for statistical analyses

For peripapillary RNFL analysis, the average RNFL thickness, 4 quadrants (superior (S), nasal (N), inferior (I), temporal (T),with 90 degrees each), and 4 additional sectors (superior-temporal (TS, 45 to 90 degrees), nasal-superior (NS, 90 to 135 degrees),nasal-inferior (NI, 225 to 270 degrees), and inferior-temporal (TI, 270 to 315 degrees) were recorded separately (Fig. 2).

### Statistical analysis

SPSS software version 21.0 for Microsoft Windows (IBM Inc., Chicago, USA) was used for statistical analysis. All data were expressed as the mean ± standard error. Independent sample t and chi-square tests were used to determine significant differences between the groups for continuous and categorical variables, respectively. The Pearson correlation test was used to detect the association between posterior pole macular thickness, RNFL thicknesses, IOP and age. Results with *p* values of less than 0.05 were considered statistically significant.

## Results

Out of 65 subjects in this investigation, we excluded five subjects due to the poor fixation and poor measurement quality. Subsequently, 60 eyes were enrolled in this study, with 30 eyes in the control group and 30 eyes in the PACS group. The mean age, sex distribution, refractive error, IOP, and axial length of all the participants were shown in Table 1. No significant difference was found in both groups.

**Table 1:** Demographic characteristics of the participants

	PACS group (*n* = 30)	Control group (*n* = 30)	P Value
Age, year	56 ± 9.24	55 ± 10.16	0.121
Sex, n (%)			
Male	1	5	
Female	29	25	0.085*
Refractive error (D)	0.91 ± 0.89	0.38 ± 0.73	0.079
VF MD, decibels	−0.98 ± 0.61	−1.31 ± 0.54	0.2
VF PSD, decibels	1.58 ± 0.26	1.61 ± 0.19	0.79
VF VFI, %	99.0 ± 0.0	99.0 ± 0.0	1.0
Intraocular Pressure (mmHg)	15.44 ± 2.50	14.49 ± 3.19	0.306
Axial length (mm)	22.37 ± 0.78	23.15 ± 0.75	0.555

Values are expressed as mean ± SD

*Chi-square test.

The PACS group exhibited significantly thinner macular retinal thickness and larger asymmetry on posterior pole region compared with the normal control group (Table 2). However, no significant difference in peripapillary RNFL parameters was found between PACS group and normal control group (Table 3).

**Table 2:** Mean posterior pole retinal thickness of total, superior, inferior, four sub-region fields and the intraocular RTA measurements (showed as Mean ± SD)

	PACS group (*n* = 30)	Control group (n = 30)	P Value
Total macular thickness	283.76 ± 10.74	293.13 ± 12.55	0.003
Superior macular thickness	285.06 ± 10.89	293.16 ± 13.15	0.012
Inferior macular thickness	282.46 ± 11.53	293.10 ± 12.45	0.001
Region 1	300.64 ± 13.82	310.79 ± 13.36	0.005
Region 2	322.54 ± 12.51	330.04 ± 14.00	0.033
Region 3	282.64 ± 10.70	291.41 ± 13.28	0.007
Region 4	265.40 ± 11.57	275.96 ± 13.31	0.002
the intraocular RTA	5.40 ± 4.09	3.53 ± 2.47	0.037

**Table 3:** Mean peripapillary RNFL thickness measurements of global and sector region (showed as (Mean ± SD) (the global thickness (G) value, the superior (S), inferior (I),nasal(N) and temporal, (T) thickness values from the 4-sector output graph; and the other supero-nasal (NS), supero-tempora (TS), infero-temporal (TI) and infero-nasal(NI) from the 7-sector output graph for statistical analyses)

	PACS group (*n* = 30)	Control group (*n* = 30)	P Value
Global RNFL thickness	115.33 ± 28.27	110.10 ± 25.43	0.465
Superior RNFL thickness	122.92 ± 37.91	117.13 ± 36.43	0.559
Temporal RNFL thickness	77.00 ± 20.85	81.10 ± 15.50	0.400
Iinferior RNFL thickness	134.74 ± 43.67	129.56 ± 39.76	0.641
Nasal RNFL thickness	93.29 ± 48.92	85.66 ± 49.75	0.563
Supero-tempora RNFL thickness	154.07 ± 50.81	140.33 ± 18.59	0.172
Supero-nasal RNFL thickness	136.03 ± 52.18	117.93 ± 40.83	0.148
Infero-temporal RNFL thickness	152.66 ± 23.96	165.90 ± 31.95	0.085
Infero-nasal RNFL thickness	140.25 ± 47.33	123.10 ± 43.18	0.158

*RNFL* Retinal nerve fiber layer

Values are expressed as mean ± SD. All values expressed in micrometers

There was no significant correlation between the total retinal thickness of PACS group with respect to the mean SE (spherical equivalent), IOP and peripapillary RNFL thickness (*p* > 0.05). A negative correlation was observed between the total retinal thickness on posterior pole region and age when all the PACS participants were analyzed (r = − 0.487, *p* = 0.006).

## Discussion

This study showed that those characteristic glaucomatous damage indicators including peripapillary RNFL thickness and IOP were similar in PACS patients and healthy controls. However, the posterior pole retinal thickness measurements obtained by Heidelberg Spectralis SD-OCT using PPAA showed significant thinner change and larger asymmetry in PACS group than normal controls. Only age had negative relation to PACS posterior pole retinal thickness.

The development of OCT makes it easy to detect glaucoma at early ages. Macular thickness was used for early diagnosis of glaucoma. Yet studies showed that macular thickness had lower diagnostic capability compared with peripapillary RNFL thickness in early glaucoma [10–13]. The reason may be that in contrast to peripapillary RNFL, macular thickness also included thicknesses of outer retinal layers. PPAA was another tool for diagnosing glaucoma and was thought to improve the accuracy of diagnosing early glaucoma. Its diagnostic accuracy was similar to that of RNFL thickness scans [14–17]. Asrani et al. [8] acquired retinal thickness of the central 20° of the posterior pole and used it in glaucoma diagnosis. Nakatani et al. [18] found that posterior pole macular measurement had high accuracy and reproducibility in diagnosing early glaucoma. Um et al. [17] reported better diagnostic sensitivity of macular hemifield thickness asymmetry in eyes with early-stage glaucoma. Sullivan-Mee et al. [19] believed that PPAA performed well in identifying early POAG compared with RNFL thickness.

In present study, we found that all the PPAA parameters including total and hemifield and four sub-regions retinal thickness measurements were generally thinner in the PACS group than that in the control group, while peripapillary RNFL thickness values were similar in both groups. Interestingly, the mean intraocular retinal thickness asymmetry in PACS group was significantly larger than control group(Fig. 2). Our explain was that PPAA acquired retinal thickness of the central 20° of the posterior pole which provide more wide and useful information. Therefore it was possible for PPAA to detect more localized change on retina [14]. Since RNFL thickness always remained the same during PACS stage compared with normal subjects, we may deduce that at PACS stage, retinal thickness of the posterior pole retina and the mean intraocular retinal thickness asymmetry might have some glaucoma -associated changes which could only be detected by PPAA.

The participants in both groups had similar IOP, axial length and spherical equivalent measurements. The normal peripapillary RNFL characteristics, in which regional thickness decreased in the order inferior > superior > nasal > temporal, were found in both groups. Since no significant difference was found between two groups in RNFL measurement, it may indicate the superiority of PPAA over peripapillary RNFL thickness analysis in early diagnosis of PACS.

This study had some limitations. Firstly, the sample size was small. Since no research was related to the database values of PACS patients for the Spectralis OCT, further study with larger samples are needed. Secondly, we didn’t measure the thickness of macular ganglion cell complex (GCC), which was reported to be affected during the glaucoma [20, 21]. Last, it would be more useful to have other parameters to expand the research.

## Conclusions

In conclusion, posterior pole retinal thickness measurements obtained by Heidelberg Spectralis SD-OCT using PPAA showed significant thinner change in PACS group than healthy controls. Additionally, IOP, axial length, spherical equivalent and peripapillary RNFL measurements were all similar in both groups. Only age had negative correlation to total retinal thickness on posterior pole region.

## Abbreviations

IOP: Intraocular pressure
PACD: Primary angle-closure disease
PACG: Primary angle-closure glaucoma
PACS: Primary angle-closure suspects
PPAA: Posterior pole asymmetry analysis
RGCs: Retinal ganglion cells
RNFL: Peripapillary retinal nerve fiber layer
SD-OCT: Spectral domain optical coherence tomography
